# Src Homology-2 Domain-Containing Protein Tyrosine Phosphatase (SHP) 2 and p38 Regulate the Expression of Chemokine CXCL8 in Human Astrocytes

**DOI:** 10.1371/journal.pone.0045596

**Published:** 2012-09-21

**Authors:** Manmeet K. Mamik, Anuja Ghorpade

**Affiliations:** Department of Cell Biology and Anatomy, University of North Texas Health Science Center, Fort Worth, Texas, United States of America; University of Nebraska Medical Center, United States of America

## Abstract

CXCL8, one of the first chemokines found in the brain, is upregulated in the brains and cerebrospinal fluid of HIV-1 infected individuals suggesting its potential role in human immune deficiency virus (HIV)-associated neuroinflammation. Astrocytes are known to be the major contributors to the CXCL8 pool. Interleukin (IL)-1β activated astrocytes exhibit significant upregulation of CXCL8. In order to determine the signaling pathways involved in CXCL8 regulation in astrocytes, we employed pharmacological inhibitors for non-receptor Src homology-2 domain-containing protein tyrosine phosphatase (SHP) 2 and mitogen-activated protein kinases (MAPK) pathway and observed reduced expression of CXCL8 following IL-1β stimulation. Overexpression of SHP2 and p38 enzymes in astrocytes led to elevated CXCL8 expression; however, inactivating SHP2 and p38 with dominant negative mutants abrogated CXCL8 induction. Furthermore, SHP2 overexpression resulted in higher SHP2 and p38 enzyme activity whereas p38 overexpression resulted in higher p38 but not SHP2 enzyme activity. Phosphorylation of SHP2 was important for phosphorylation of p38, which in turn was critical for phosphorylation of extracellular signal regulated kinase (ERK). Thus, our findings suggest an important role for SHP2 in CXCL8 expression in astrocytes during inflammation, as SHP2, directly or indirectly, modulates p38 and ERK MAPK in the signaling cascade leading to CXCL8 production. This study provides detailed understanding of the mechanisms involved in CXCL8 production during neuroinflammation.

## Introduction

Chemokines, or chemotactic cytokines, have the ability to recruit leukocyte subsets into sites of inflammation. CXCL8, formerly called interleukin (IL)-8, was identified as the first member of a still growing chemokine family [1]. Besides attracting neutrophil subsets into sites of inflammation by chemotaxis, CXCL8 can also activate monocytes and T cells. In this study, we focused on chemokine CXCL8 based on recent research implicating this chemokine in neuropathogenesis during several neurodegenerative disorders such as Alzheimer’s disease and human immune deficiency virus (HIV)-1 infection [2], [3]. Chemokine CXCL8 levels are elevated in serum, lymphoid tissue, plasma and cerebrospinal fluid (CSF) of HIV-1 infected individuals [4], [5]. CXCL8 is produced and released in the brain microenvironment by a variety of cells including microglia and astrocytes [6], [7]. Together, immune cells and immune mediators, which are comprised of cytokines and chemokines, contribute to the disruption of neuronal homeostasis leading to neurodegeneration [8]. The effect of CXCL8 on neurons is a major area of interest. Research in our laboratory has shown that CXCL8 protects human neurons from amyloid-β-induced neurotoxicity [9]. We also reported that astrocytes transfected with an HIV-1_YU-2_-expressing plasmid, demonstrated elevated CXCL8 [10]. Sources of elevated CXCL8 include wide variety of cells including activated microglia and astrocytes in the brain [11], [12]. Astrocytes are the major cell type of central nervous system (CNS), and are known to secrete CXCL8 in response to inflammation. However, the regulatory mechanisms of CXCL8 production in astrocytes are not well-defined. Since CXCL8 is a potential mediator of neutrophil-induced inflammation, in this study we investigated the underlying astrocytic signaling networks involved in chemokine CXCL8 production.

Understanding of the inflammatory responses of astrocytes is of particular importance to unravel the process of neuropathogenesis in HIV-associated dementia (HAD) and several neurodegenerative diseases. CXCL8 is expressed by astrocytes in HIV-1 encephalitic tissue and is upregulated in an astrocytic cell line infected with HIV-1 [13], [14]. This chemokine also stimulates HIV-1 replication in macrophages and T-cells [4]. Thus, CXCL8 upregulation by activated astrocytes contributes to the inflammatory disease process. However, the mechanisms of CXCL8 production are not completely understood in human astrocytes.

Src homology-2 domain-containing protein tyrosine phosphatase (SHP) 2 (also known as PTPN11, PTP1D, SHPTP-2) is a ubiquitously expressed cytoplasmic protein tyrosine phosphatase (PTP) which acts downstream of many tyrosine kinases and cytokine receptors (reviewed in [15]). SHP2 in its native form is autoinhibited by N-terminal SH2 domains blocking the active site of the enzyme. Its catalytic activation requires phosphorylation at specific residues, which opens the conformation and relieves the autoinhibition. It is reported that following epidermal growth factor or platelet-derived growth factor stimulation, the growth factor receptors bind to the SHP2 N-terminal SH2 domains, which in turn binds to Grb2-Sos and leads to Ras/mitogen-activated protein kinases (MAPK) activation. MAPK are a family of serine/threonine kinases comprising of extracellular signal regulated kinase (ERK), p38 and c-jun N-terminal kinases. SHP2 is known to activate ERK in human fibroblasts; however, SHP2 is implicated to act downstream or parallel to the Ras/MAPK pathway [16]. Overexpression of catalytically inactive form of SHP2 (SHP2CS) is shown to exert dominant negative effect on Ras/MAPK stimulation in different cellular models [17]. Phosphorylation and activation of ERK and p38 is reported when mixed glial cells are activated with IL-1β [18]. While strong evidence supports involvement of SHP2 in the MAPK pathway in other cells, it has never been reported in astrocytes and the mechanisms involved are still unclear.

In the present study, we hypothesized that SHP2 and MAPK participate in the upregulation of astrocyte CXCL8 expression following stimulation with HIV-1-relevant stimuli such as IL-1β. We propose that IL-1β receptors directly or indirectly lead to phosphorylation or activation of SHP2, which in turn modulates MAPK to increase CXCL8 expression in astrocytes. In this report, we further delineated the order in which SHP2 and p38/ERK MAPK regulate CXCL8 expression in activated astrocytes. Our data demonstrates that CXCL8 is upregulated in HIV-1 infected individuals and that astrocytes produce elevated levels of CXCL8 following activation by proinflammatory stimuli. We show for the first time that SHP2 mediates p38 MAPK signaling to regulate the expression of chemokine CXCL8 in human astrocytes. The data presented in this study provide insights into the regulation of CXCL8 in astrocytes during inflammation, which may provide means to therapeutically modulate the levels of this chemokine.

## Methods

### Preparation of Human Brain Tissue Extracts

Human brain specimens from the frontal cortex were provided by the National NeuroAIDS Tissue Consortium, Center for Neurovirology and Neurodegenerative Disorders brain bank and Rapid Autopsy Program at the University of Nebraska Medical Center [19]. The institutional review boards of both the Universities of Nebraska Medical Center and North Texas Health Science Center approved the collection of human tissues for research. All donors gave informed written consent, which permits research use of their tissues and informed them of possible conflicts of interest. Brain samples are from the cohort as described in [20]. Briefly, the HIV-1 infected patients included seven males and four females between the ages of 25 and 55 years; the age-matched control donors included two males and three females between the ages of 37 and 52 years. Brain lysates and RNA were isolated as previously described [21]. Protein concentration was determined by bicionconic acid method as suggested by the manufacturer (Pierce, Rockford, IL, USA).

### Isolation and Cultivation of Primary Human Astrocytes

Human astrocytes were isolated from first- and early second-trimester aborted specimens, ranging from 82 to 127 days, obtained from the Birth Defects Laboratory, University of Washington, Seattle, WA in full compliance with the ethical guidelines of the NIH. The institutional review boards of both the Universities of Washington and North Texas Health Science Center approved the collection of human tissues for research. The Birth Defects Laboratory obtained written consent from all tissue donors. Human astrocytes were isolated as previously described in [22]. Briefly, brain tissues were dissected and mechanically dissociated. Cell suspensions were centrifuged, suspended in media, and plated at a density of 20×10^6^ cells/150 cm^2^. The adherent astrocytes were treated with trypsin and cultured under similar conditions to enhance the purity of replicating astroglial cells. The astrocyte preparations were routinely >99% pure as measured by immunocytochemistry staining for glial fibrillary acidic protein and microglial marker CD68 determine possible microglial contamination and contribution of microglia in inflammatory responses.

### RNA Extraction and Gene Expression Analyses

RNA was isolated (as described in [23]) from astrocytes treated as described in subsequent sections and gene expression was assayed by real-time PCR using StepOnePlus (Life Technologies, Carlsbad, CA). Commercially available TaqMan® gene expression assays were used to measure CXCL8 (Life Technologies, Cat# Hs00174103_ml) and glyceraldehyde 3-phosphate dehydogenase (GAPDH) (Cat# 4310884E) mRNA levels. GAPDH, a ubiquitously expressed housekeeping gene, was used as an internal normalizing control. The 30 µl reactions were carried out at 48°C for 30 min, 95°C for 10 min, followed by 40 cycles of 95°C for 15 s and 60°C for 1 min in 96-well optical, real-time PCR plates. Samples were analyzed in triplicates. Relative quantities were determined by comparison to an experimental standard curve with known cDNA quantities. Gene expression is expressed as relative quantity of CXCL8 normalized to that of GAPDH.

### Astrocyte Treatment and Activation

Primary astrocytes were treated with or without p38 inhibitors: SB203580 and SB202190 (20 µM, Santa Cruz Biotechnology, Santa Cruz, CA); ERK inhibitors: PD98059 and U0126 (20 µM, Sigma Aldrich Inc., St Louis, MO); SHP2 inhibitor: phenylhydrazonopyrazolone sulfonate (PHPS1) (20 µM, Sigma); or PTP inhibitor: sodium orthovanadate (Na_3_VO_4_) (1 mM, Sigma) for 2 h prior to activation with IL-1β (20 ng/ml, R&D Systems, Minneapolis, MN), as previously described [10], [24], [25]. This dose of IL-1β is well within the range of 5–100 ng/ml currently used to activate astrocytes [26] and levels induced in animal models [27], [28].

### Plasmid Constructs and Transfection into Astrocytes

Overexpression vectors were obtained from the non-profit plasmid repository, Addgene, Cambridge, MA. The SHP2 overexpression constructs: wild-type SHPWT (Addgene plasmid 8381) and dominant negative mutant SHP2CS (Addgene plasmid 8382) were deposited by Ben Neel [29]; and the p38 overexpression constructs: p38 (Addgene plasmid 20351) and dominant negative mutant p38agf (Addgene plasmid 20352) were deposited by Roger Davis [30]. Astrocytes were transfected using the P3 primary cell kit, nucleofector device and shuttle attachment (Lonza Inc., Walkersville, MD, USA) as previously standardized [10]. Briefly, astrocytes were suspended in nucleofector solution and plasmid constructs (1 µg/1.5 million cells). Transfected cells were supplemented with astrocyte media and incubated for 30 min at 37°C prior to plating. Twelve to 24 h post-plating, cells were washed and treated as described above.

### Quantification of CXCL8 by ELISA

CXCL8 protein levels were determined from culture supernatants by a CXCL8 specific sandwich ELISA (R&D Systems, Cat# S800C) according to manufacturer’s protocol. Absorbance was determined by Spectromax M5 microplate reader using SoftMax Pro V5 software (Molecular Devices, Sunnyvale, CA).

### Determination of *in vitro* Phosphatase Activity

SHP2 phosphatase activity was measured in whole cell protein lysates using SHP2 assay kit (R&D Systems, Cat# DYC2809). Briefly, transfected astrocytes grown to confluence in tissue culture flasks (8 million cells/75 cm^2^ flask) were scraped and suspended in M-PER mammalian protein extraction reagent with protease inhibitors (Thermo Fisher Scientific, Rockford, IL). Cell lysates containing 100 ng/ml of total protein were immunoprecipitated with SHP2-specific beads, incubated with a synthetic phosphopeptide as substrate and phosphate released was measured. Absorbance of the reaction mixture was measured at 620 nM.

### Determination of *in vitro* Kinase Activity

p38 kinase activity was measured in whole cell protein lysates using p38 MAP kinase assay kit (Cell Signaling, Cat# 9820). Briefly, 50 µg/ml of total protein were incubated with immobilized phosphorylated (P)-p38 primary antibody overnight at 4°C. After elution with kinase buffer, eluent was incubated with ATP and activating transcription factor (ATF)-2 as substrate, for 30 min at 30°C. Phosphorylation of ATF-2 is proportional to p38 kinase activity, which was visualized by western blot analysis as described below.

### Western Blot Analysis

Equal amounts of protein samples (25 µg) were boiled with 4X NuPAGE loading sample buffer (Life Technologies) for 5–10 min, resolved by NuPage 4–12% Bis tris gel and subsequently transferred to a nitrocellulose membrane using i-Blot (Life Technologies, Carlsbad, CA, USA). The membrane was incubated with individual primary antibodies (Cell Signaling; SHP2 #3752, P-SHP2 #3751, p38 #9212 and P-p38 #9216) at a dilution of 1∶1000 overnight at 4°C, washed and then incubated with anti-mouse or anti-rabbit goat antibody IgG conjugated to horseradish peroxidase (1∶10,000, Bio-Rad) for 2 h at room temperature. The membrane was then developed with SuperSignal west femto substrate (Thermo) in a Fluorochem HD2 Imager (ProteinSimple, Inc. Santa Clara, CA). β-actin (1∶1,000, Cell Signaling) immunoblotting was used as a loading control.

### Determination of Astrocyte Metabolic Activity

Following experimental manipulations described above, five percent (3-(4,5-dimethylthiazol-2-yl)-2,5-diphenyltetrazolium bromide (MTT) reagent in astrocyte medium was added to astrocytes and incubated for 20–45 min at 37°C. MTT is metabolically reduced to purple formazan crystals by living cells. The MTT solution was removed and crystals were dissolved in DMSO for 15 min with gentle agitation. The absorbance of the DMSO/crystal solution was assayed at 490 nm, as previously described [31].

### Statistical Analyses

Statistical analyses were carried out using Prism V5.0 (GraphPad Software, La Jolla, CA) with one-way analysis of variance (ANOVA) and Newman-Keuls post-test for multiple comparisons Student’s t-test was performed for paired observations. Significance was set at p<0.05 and data represents means +/− standard error of the mean (SEM). Data presented is representative of a minimum of three independent experiments with two or more independent donors.

## Results

### HIV-1 Infection Elevates Chemokine CXCL8 Levels in Human Brain

**Figure 1:**
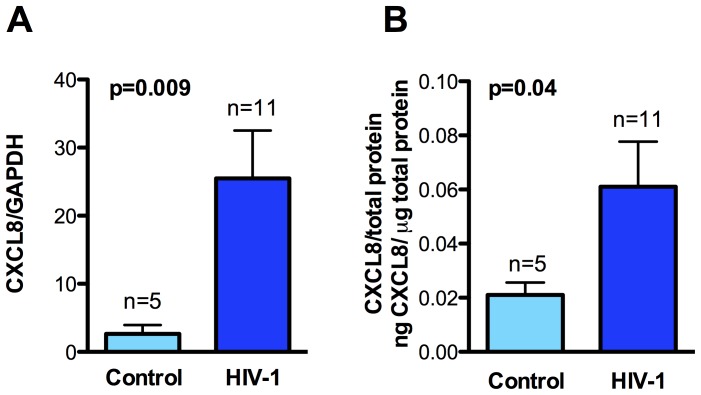
CXCL8 levels are elevated in brain lysates of HIV-1 infected patients. Human brain mRNA and protein lysates collected from frontal cortex of HIV-1 infected individuals and age matched controls were analyzed for CXCL8 levels. **A)** CXCL8 mRNA expression analyzed by real-time PCR normalized to GAPDH. **B)** CXCL8 protein levels assayed by ELISA shown as ng/µg total protein. Results are expressed as mean ± SEM of indicated number of donors. Statistical analyses were performed using unpaired student’s t-test.

Chemokine CXCL8 levels are upregulated in CSF of HAD patients when compared to HIV-1 infected individuals [3]. We examined CXCL8 levels in brain lysates isolated from frontal cortex of eleven HIV-1 infected patient brains and five age-matched controls. CXCL8 levels, as measured in mRNA by RT-PCR and protein lysates by ELISA, were higher in samples obtained from HIV-1 infected patients at both mRNA and protein levels, as compared to control samples. The mRNA levels in HIV-1 infected patients (mean 25.48) exhibited a 9.5-fold increase when compared to control samples (mean 2.66), with the lowest value observed in HIV-1 infected group being greater than the highest value in control samples (Figure 1A, p = 0.009). In accordance with increased mRNA levels, a 2.9 fold increase in CXCL8 protein levels was measured in HIV-1 infected brain lysates (mean 0.06103) as compared to controls (mean 0.02102) expressed as ng/µg of total protein (Figure 1B, p = 0.04).

### Proinflammatory Cytokines Increase Astrocyte CXCL8 Expression

**Figure 2:**
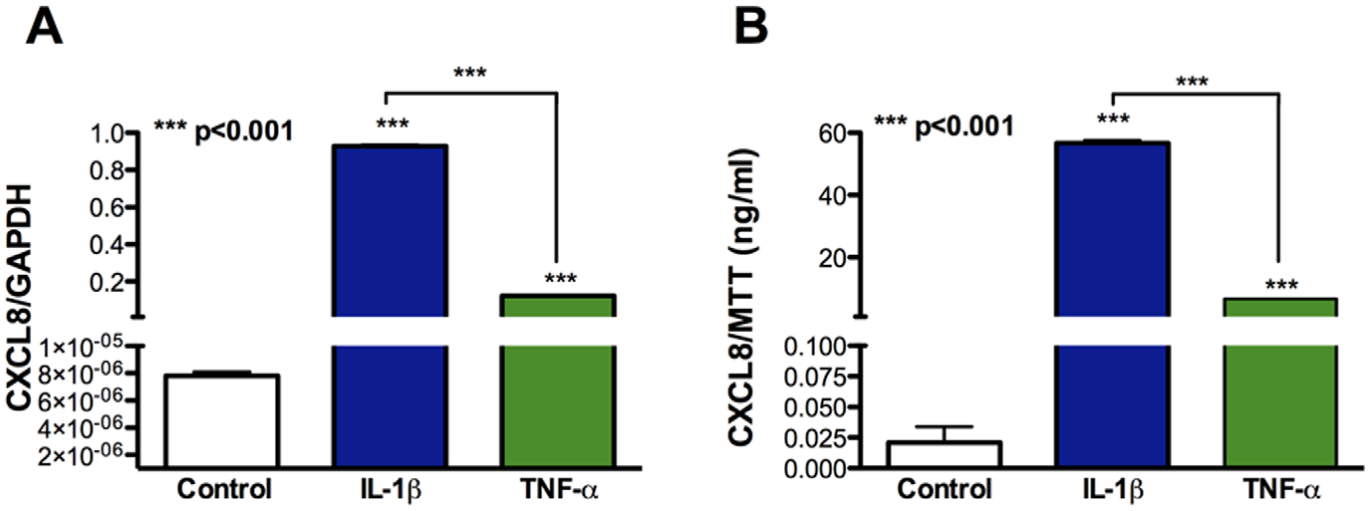
Increased CXCL8 expression in astrocytes activated by IL-1β and TNF-α. Cultured human astrocytes were treated with IL-1β (20 ng/ml) or TNF-α (20 ng/ml). **A)** CXCL8 mRNA expression 8 h post-activation normalized to GAPDH determined by real-time PCR. **B)** CXCL8 protein levels 24 h post-activation normalized to MTT determined by ELISA. Results are representative of three independent experiments performed in triplicates. Results are expressed as mean ± SEM, analyzed by one-way ANOVA and Newman-Keuls post-test for multiple comparisons.

Since astrocytes are the most abundant cell in the CNS, we next examined whether they are a significant source of CXCL8. During HIV-1 infection in the brain, proinflammatory cytokines such as IL-1β and tumor necrosis factor (TNF)-α are released by infiltrating HIV-1 infected macrophages [32]. To study the effect of these cytokines on CXCL8 production, astrocytes were treated with IL-1β and TNF-α (20 ng/ml). RNA was isolated at 8 h and supernatants were collected 24 h post-treatment. While control astrocytes showed low or undetectable levels of CXCL8, a robust increase in CXCL8 levels was observed following treatment with IL-1β (90,000-fold) or TNF-α (10,000-fold). CXCL8 levels increased significantly at both RNA and protein levels indicating release of CXCL8 by stimulated astrocytes **(** **Figure 2** **, p<0.001)**. Secreted CXCL8 protein levels also increased in a dose-dependent manner upon astrocyte activation with either IL-1β (0.1 to 100 ng/ml) or TNF-α (1 to 100 ng/ml), as assayed by ELISA (data not shown). Taken together, stimulation of astrocytes by proinflammatory cytokines leads to release of chemokine CXCL8.

### IL-1β-mediated Increase in CXCL8 is SHP2 and MAPK Dependent

**Figure 3:**
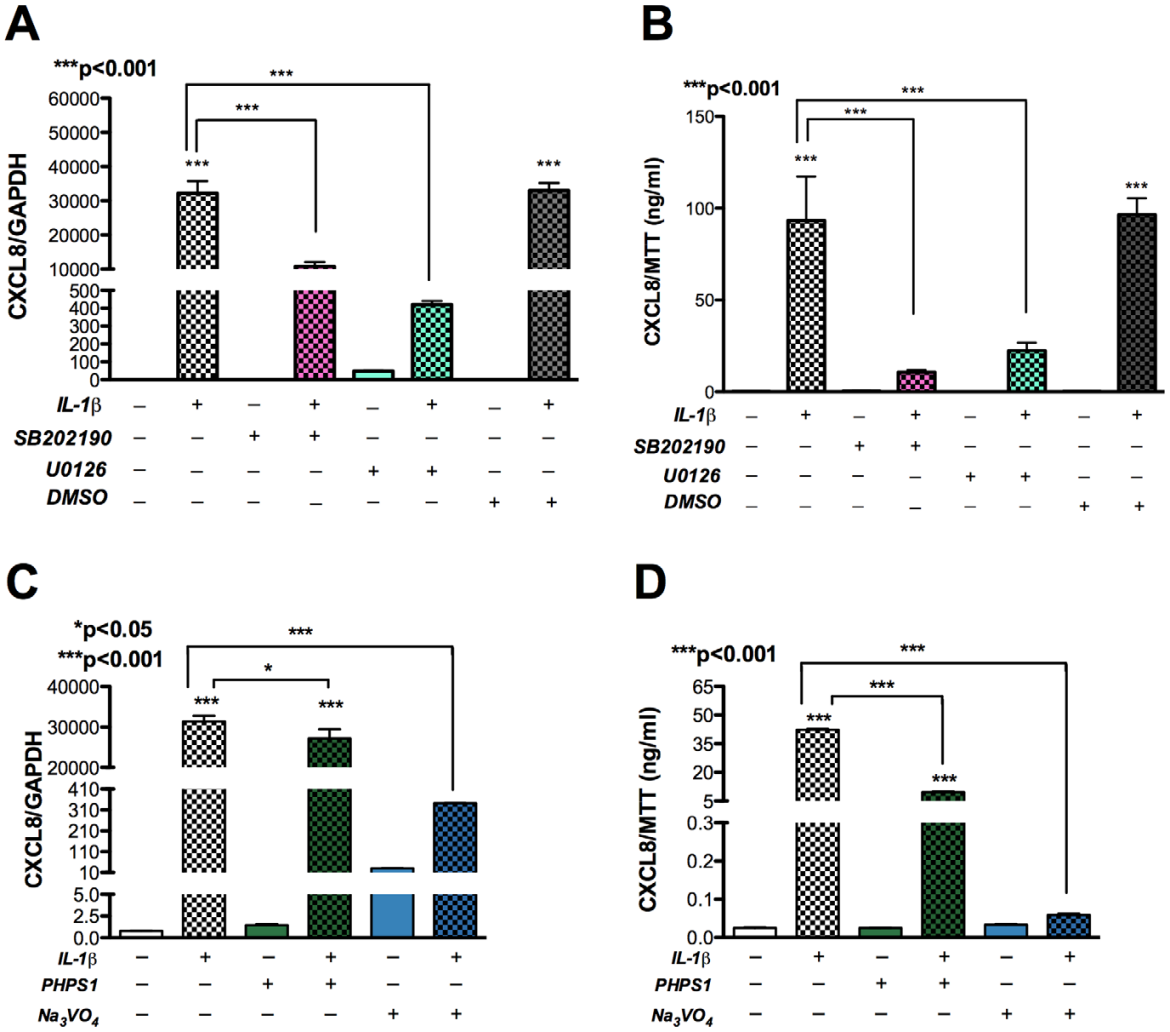
MAPK and SHP2 regulate IL-1β-mediated increased CXCL8 expression in astrocytes. Cultured human astrocytes were pretreated with MAPK and SHP2 inhibitors for 2 h, followed by activation with IL-1β (20 ng/ml). Untreated controls were maintained in parallel. RNA was isolated 8 h post-activation and supernatants were collected 24 h post-activation. CXCL8 mRNA was measured using real-time PCR, while protein expression was measured by ELISA. **A)** CXCL8 mRNA levels normalized to GAPDH after pretreatment with inhibitors specific to p38, SB202190 and ERK, U0126. **B)** CXCL8 protein levels normalized to MTT after pretreatment with inhibitors specific to p38, SB202190 and ERK, U0126. DMSO was used as a solvent control. **C)** CXCL8 mRNA levels normalized to GAPDH after pretreatment with inhibitors specific to SHP2, PHPS1 and PTP, Na_3_VO_4_. **D)** CXCL8 protein levels normalized to MTT after pretreatment with inhibitors specific to SHP2 (PHPS1) or PTP (Na_3_VO_4_). Results are representative of three independent experiments performed in triplicate and expressed as mean ± SEM, analyzed by one-way ANOVA and Newman-Keuls post-test for multiple comparisons.

To investigate signaling pathways involved in astrocyte CXCL8 production following activation, we used a panel of pharmacological inhibitors against SHP2 and MAPK (p38 and ERK). MAPK are implicated in CXCL8 gene regulation during inflammation [33]. Since we observed significant upregulation in CXCL8 expression in IL-1β-activated astrocytes, we evaluated IL-1β-mediated CXCL8 expression during independent inhibition of SHP2, p38 and ERK pathways. While basal CXCL8 levels remained unchanged following pretreatment of astrocytes with SB202190 (p38 inhibitor), and U0126 (ERK inhibitor), each of the inhibitors significantly reduced the induction of CXCL8 mRNA and protein expression by IL-1β. Dimethyl sulphoxide (DMSO) was used as a solvent control **(** **Figure 3A, B** **; p<0.001).** Another ERK inhibitor PD98059 was also employed in the experiment and yielded significant decrease in CXCL8 mRNA and protein levels in astrocytes treated with IL-1β (data not shown). Similarly, pretreatment of astrocytes with Na_3_VO_4_ (PTP inhibitor) and PHPS1 (SHP2 inhibitor) led to significant inhibition of the IL-1β-mediated increase in CXCL8 mRNA and protein levels **(** **Figure 3C, D** **; p<0.001).** Thus SHP2, p38 and ERK were each implicated in modulation of CXCL8 levels in IL-1β-activated astrocytes. These results suggested that both SHP2 and MAPKs regulate CXCL8 mRNA and protein expression in activated astrocytes, either as part of one signaling cascade or independently in different signaling cascades eventually converging to CXCL8 expression.

Next, we employed overexpression of wild-type and dominant negative SHP2 and p38 in order to elucidate the interaction of these two enzymes in regulation of CXCL8. We overexpressed SHP2 and inhibited p38, and vice versa, to understand whether SHP2 and p38 were part of the same signal transduction cascade leading to CXCL8 expression in astrocytes.

### Overexpression of SHP2 and p38 in Astrocytes Increases CXCL8 Production

**Figure 4:**
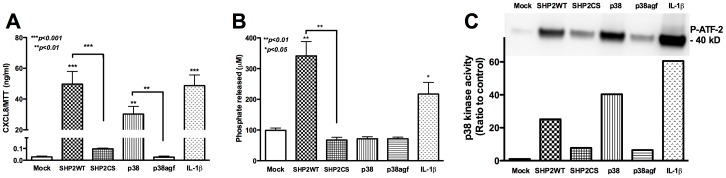
CXCL8 upregulation by SHP2 and p38 overexpression in astrocytes. Primary human astrocytes were transfected with SHP2WT and p38 overexpression plasmids with corresponding dominant negative mutants SHP2CS and p38agf. Twenty-four h post-transfection supernatants were analyzed for CXCL8 protein levels by ELISA. 24 h post-transfection astrocytes were treated with IL-1β (20 ng/ml) for 24 h, protein lysates and supernatants were collected and analyzed for SHP2 and p38 enzyme activity. **A)** CXCL8 protein levels assayed by ELISA normalized to MTT. **B)** SHP2 *in vitro* phosphatase assay showing phosphate released as a measure of SHP2 phosphatase activity. Results are representative of three independent experiments performed in triplicate and expressed as mean ± SEM, analyzed by one-way ANOVA and Newman-Keuls post-test for multiple comparisons. **C)** *In vitro* p38 kinase assay based on immunoprecipitation of p38, followed by incubation with ATF-2 as substrate. P-ATF-2 band at 40 kD, which is a function of higher p38 kinase activity, is shown with densitometry. Results are representative of three independent experiments.

To further validate the role of SHP2 and p38 MAPK in astrocyte CXCL8 production, we transiently transfected wild-type SHP2 (SHP2WT) and p38 overexpression constructs into astrocytes. The dominant negative mutants SHP2CS and p38agf were transfected into astrocytes, in parallel, for comparison. CXCL8 protein levels were measured by ELISA in astrocyte culture supernatants 24 h post-transfection. Interestingly, we found significantly increased CXCL8 protein levels in SHP2WT- and p38-transfected astrocytes as compared to dominant negative controls and mock (**Figure 4A**, p<0.001 and p<0.01, respectively)**.** Increased CXCL8 protein in astrocyte supernatants resulted from SHP2 and p38 overexpression, even in the absence of IL-1β stimulation. Supernatants from IL-1β-activated astrocytes were used as a positive control, demonstrating a 2400-fold induction in CXCL8 protein levels similar to that shown during SHP2WT overexpression, as compared to 1500-fold during p38-overexpression **(** **Figure 4A** **)**. *In vitro* SHP2 phosphatase assay, a measure of SHP2 function in whole cell protein lysates, showed high SHP2 enzyme activity in SHP2WT-transfected astrocytes as compared to mock- and SHP2CS-transfected indicating efficient transfection, expression and activity of SHP2 protein. Concurrently, SHP2 phosphatase activity increased by two-fold following IL-1β-activation of mock-transfected astrocytes **(** **Figure 4B** **, p<0.05)**. Thus, IL-1β stimulation led to elevated CXCL8 expression in astrocytes in a SHP2-dependent manner. Similarly, p38 kinase activity in astrocytes was measured by *in vitro* p38 kinase assay following transfection. As expected, p38 kinase activity in p38-transfected astrocytes increased by 40-fold, indicating efficient transfection, expression and activity **(** **Figure 4C** **)**. Furthermore, p38 kinase activity was evident in IL-1β-activated astrocytes, demonstrating CXCL8 expression is regulated in p38-dependent manner in IL-1β-activated astrocytes. In comparison, p38agf-transfected astrocytes exhibited basal p38 enzyme activity, similar to that of mock-transfected astrocytes **(** **Figure 4C** **)**. The dominant negative mutants expressed the corresponding inactive enzymes efficiently. Surprisingly, we observed increased levels (∼25-fold) of active p38 kinase in SHP2WT-transfected astrocytes, thus elucidating the order in which the two enzymes act in regulation of astrocyte CXCL8 expression.

### p38 Acts Downstream of SHP2-mediated Increase in Astrocyte CXCL8 Expression

**Figure 5:**
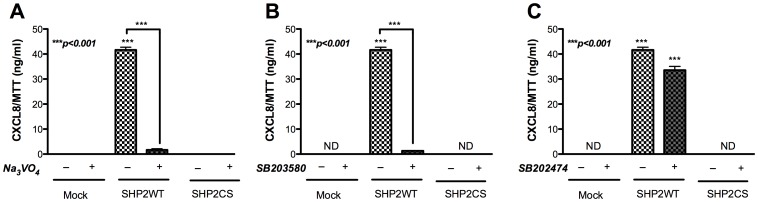
Effect of SHP2 overexpression and p38 inhibition on CXCL8 levels. SHP2WT- and SHP2CS-transfected astrocytes were treated with PTP inhibitor, Na_3_VO_4_; p38 inhibitor, SB203580, or negative control inhibitor, SB202474. CXCL8 levels measured by ELISA in cellular supernatants collected 24 h post-treatment with **A)** Na_3_VO_4_ **B)** SB203580 **C)** SB202474. ND denotes not detectable. Results are representative of three independent experiments performed in triplicate and expressed as mean ± SEM, analyzed by one-way ANOVA and Newman-Keuls post-test for multiple comparisons or student’s t-test.

**Figure 6:**
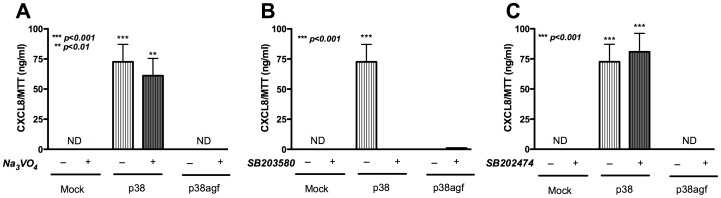
Effect of p38 overexpression and SHP2 inhibition on CXCL8 levels. p38- and p38agf-transfected astrocytes were treated with PTP inhibitor, Na_3_VO_4_; p38 inhibitor, SB203580, or negative control inhibitor, SB202474. CXCL8 levels measured by ELISA in cellular supernatants collected 24 h post-treatment with **A)** Na_3_VO_4_ **B)** SB203580 **C)** SB202474. ND denotes not detectable. Results are representative of three independent experiments performed in triplicate and expressed as mean ± SEM, analyzed by student’s t-test.

p38 MAPK is known to act upstream of SHP2 in CXCL8 regulation in hepatocytes [34]. To explore the order in which the two enzymes, a phosphatase and a kinase, act to regulate astrocyte CXCL8 expression, SHP2 was overexpressed in astrocytes followed by independent inhibition of SHP2 and p38 activity using pharmacological inhibitors. The SHP2-mediated increase in CXCL8 expression remained below detection in culture supernatants of SHP2WT-transfected astrocytes in the presence of PTP and p38 inhibitors, Na_3_VO_4_ and SB203580, respectively **(** **Figure 5A, B** **)**. However, treatment with a negative control inhibitor, SB202474, did not inhibit SHP2WT-induced CXCL8 expression **(** **Figure 5C** **)**. These data indicated that p38 is important for regulation of CXCL8 protein expression in astrocytes, and SHP2 overexpression alone could not bypass p38 activation. In parallel, p38 and p38agf-transfected astrocytes were treated with Na_3_VO_4_ and SB203580. The p38-mediated CXCL8 increase was unchanged upon treatment with PTP inhibitor Na_3_VO_4_ **(** **Figure 6A** **)**; however, CXCL8 protein levels were undetectable, as expected, after treatment with p38 inhibitor SB203580 **(** **Figure 6B** **)**. The negative control inhibitor had no effect on the p38-mediated CXCL8 increase **(** **Figure 6C** **)**. Therefore, p38 was implicated as acting downstream of SHP2 in the regulation of astrocyte CXCL8 expression.

### Phosphorylation of SHP2 is Important for Downstream Phosphorylation of p38 and ERK

**Figure 7:**
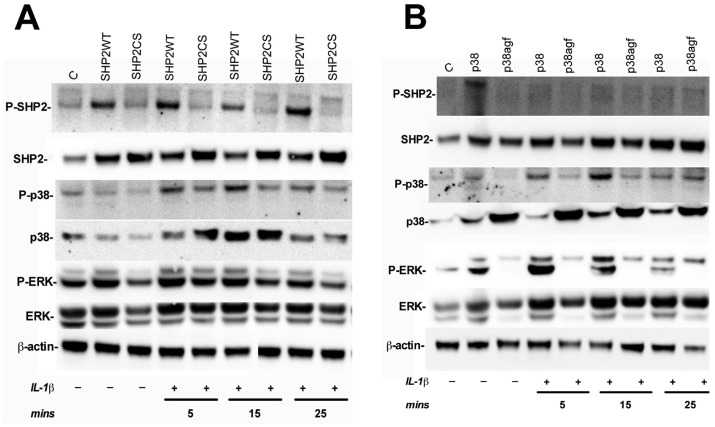
SHP2, p38 and ERK phosphorylation following IL-1β stimulation of SHP2WT-, SHP2CS-, p38- and p38agf-transfected astrocytes. Astrocytes were transfected with SHP2WT, SHP2CS, p38 or p38agf plasmids and stimulated with or without IL-1β for 5, 15 and 25 min. Whole cell protein lysates were collected and equivalent amounts were resolved by SDS-PAGE, transferred and immunoblotted for P-SHP2, SHP2, P-p38, p38, P-ERK and ERK. β-actin was used as loading control. **A)** Immunoblot of SHP2WT- and SHP2CS-transfected astrocytes following stimulation with IL-1β. **B)** Immunoblot of p38- and p38agf-transfected astrocytes following stimulation with IL-1β.

Phosphorylation of both SHP2 and p38 is important for the activation of their enzymatic functions [35]. Phosphorylation of p38 and ERK is stimulated by IL-1β in astrocytes [36], [37]. Therefore, the phosphorylation of SHP2, p38 and ERK was compared in SHP2WT-, SHP2CS-, p38- and p38agf-transfected astrocytes with or without IL-1β stimulation for 5, 15 and 25 min. Phosphorylation of SHP2, p38 and ERK increased during SHP2WT overexpression, which further increased upon IL-1β stimulation **(** **Figure 7A** **)**. Interestingly, ERK phosphorylation was evident during p38 overexpression with little to no SHP2 phosphorylation **(** **Figure 7B** **)**, suggesting that SHP2 phosphorylation regulates p38 phosphorylation, which in turn, regulates ERK phosphorylation.

## Discussion

In this study we investigated the intracellular signaling mechanisms of chemokine CXCL8 in astrocytes in the context of neuroinflammation during HIV-1 infection. CXCL8 is an important chemokine upregulated in CSF of HAD patients [3]. Consistent with prior studies, our data also showed elevated CXCL8 levels in brains of HIV-1 infected patients. We found robust increases in astrocyte CXCL8 expression in response to proinflammatory stimuli as previously reported [6]. Our study shows that SHP2, p38 and ERK are involved in regulating IL-1β-mediated increase in CXCL8 production by human astrocytes. In this study, we have identified SHP2 as an important signal transducer upstream of p38 and ERK directing upregulation of CXCL8 in activated astrocytes.

During the course of HIV-1 CNS infection, astrocytes respond to proinflammatory stimuli by release of several cytokines and chemokines, which are important mediators of HIV-1-induced neuronal damage [38]. CXCL8 is involved during innate immune responses in the CNS, and likely contributes to intercellular interactions leading to neuroinflammation. Several studies relate elevated CXCL8 levels with neurodegeneration; however, the molecular mechanisms involved are incompletely understood. Increased CXCL8 levels are observed during congenital human cytomegalovirus infection of brain vascular pericytes, which often leads to CNS abnormalities [39]. Interferon-β, an anti-inflammatory mediator, repressed CXCL8 gene expression in peripheral blood cells implicating a possible mechanism behind its anti-inflammatory properties through repression of CXCL8 [40]. Astrocyte activation and overexpression of cytokines and chemokines like IL-1β, TNF-α and CXCL8 have been associated with Alzheimer’s disease [41], [42]. We report high levels of CXCL8 in HIV-1 infected individuals, which may be a likely candidate accounting for progressive neurodegeneration associated with chronic HIV-1 infection. Elevated levels of CXCL8 have been reported after ischemic brain injury [43]. A recent study showed high levels of CXCL8 expressed by human brain endothelial cells from patients with multiple sclerosis [44]. Our results on HIV-1 infected patients’ CXCL8 levels, combined with previous reports, strongly implicate CXCL8 to be involved in neuroinflammation during variety of neurodegenerative conditions, including HIV-1 infection.

Sources of CXCL8 include activated microglia, astrocytes and endothelial cells. Neutrophils are also shown to produce CXCL8 possibly to amplify leukocyte recruitment [45]. Astrocytes are reportedly major producers of CXCL8 in the CNS during an inflammatory response [46]. In accordance with several groups, we also found upregulation in CXCL8 as an astrocytic response to proinflammatory cytokines like IL-1β and TNF-α [3], [6]. Furthermore, the increase is found at both mRNA and protein levels, suggesting the induction of astrocyte CXCL8 gene expression in response to cytokines. This observation highlights involvement of signal transduction pathways communicating between extracellular environment of astrocytes and inside of nucleus. Since astrocytes are associated with the endothelial cells in blood-brain barrier, it is likely that proinflammatory cytokines activate astrocytes, which release chemokines to attract leukocytes from the periphery into the CNS, a process that eventually leads to neuroinflammation. Another study shows elevated cytokine levels, including CXCL8, when astrocyte cell lines were cocultured with peripheral blood mononuclear cells [47].

Regulation of CXCL8 has been studied in a variety of cell types [3], [34], [48], [49]. MAPK is an important signaling pathway studied in context of CXCL8 regulation in different cell types. The observed repression of IL-1β-induced CXCL8 upregulation when p38 and ERK pathways were inhibited using specific inhibitors, clearly implicated the involvement of MAPK in astrocyte CXCL8 regulation. Since SHP2 has been studied in relation to cytokine receptors in different cellular systems, we extended this observation to astrocytes in the present study. SHP2 is a ubiquitously expressed non-receptor PTP, implicated in signaling events downstream of receptors for growth hormones and cytokines [50]. SHP2 is also known to control cell growth and differentiation [51], [52]. Mutations in the SHP2 gene are associated with genetic disorders, such as Noonan syndrome and Leopard syndrome, which are thought to result from abnormal MAPK activation [53].

We report that SHP2 acts to promote MAPK activity in activated astrocytes, which is consistent with reports in other cell systems [54], [55]. In fibroblasts, treatment with fibroblast growth factor or platelet-derived growth factor leads to SHP2 phosphorylation and binding to Grb2, facilitating ERK activation [17]. Another docking protein, Gab1, is known to associate with SHP2 in epidermal growth factor-induced ERK activation [56], [57], [58]. Our study highlights the function of SHP2 in modulating p38 activation, an important event in astrocytes for ERK activation, leading to increased expression of CXCL8 during inflammation. Although MAPKs are known to be involved in expression of certain proinflammatory genes, the upstream and downstream regulators are not well described. We show that expression of chemokine CXCL8 is significantly upregulated in astrocytes transfected with SHP2WT and p38 overexpression constructs; however, transfection with dominant negative mutants, incapable of becoming phosphorylated, did not show increased CXCL8 levels in human astrocytes. Elevated levels of CXCL8 resulted as direct effect of SHP2 and p38 enzyme function, which was evident from high phosphatase and kinase activity in the SHP2- and p38-overexpressing astrocytes.

In exploring the interaction of SHP2 with Ras/MAPK pathway in astrocytes, interestingly we found that inhibition of p38 blocked SHP2-overexpression-induced CXCL8 expression in astrocytes. In contrast, inhibition of SHP2 had no effect on p38-stimulated CXCL8 upregulation. This clearly suggested that SHP2 activity is important for p38 activity. Our interpretation of the results was strengthened by observing high p38 kinase activity in SHP2-overexpression. However, SHP2 phosphatase activity remained at basal levels during p38-overexpression. Furthermore, in SHP2-deficient cells, there was little or no phosphorylation of p38 and ERK following IL-1β stimulation; inactivation of SHP2 resulted in suppression of the P-p38 and P-ERK signals. SHP2 is a phosphatase and our results support its role in p38 and ERK phosphorylation, indicating that p38 and ERK are not direct substrates for SHP2 phosphatase activity. However, SHP2 is reported to be required for ERK activation in some, but not all growth factors and may act upstream, downstream or parallel to Ras/MAPK. A recent report shows CXCL8 production in hepatocytes in response to Hepatitis C virus and HIV-1 envelope proteins. However, p38 acts upstream to SHP2 in regulation of chemokine CXCL8 production by hepatocytes [34]. It is thus imperative to assess cellular system specific signaling mechanisms regulated by SHP2 and MAPK, and we report for the first time the involvement of SHP2 in the modulation of CXCL8 expression in human astrocytes.

**Figure 8:**
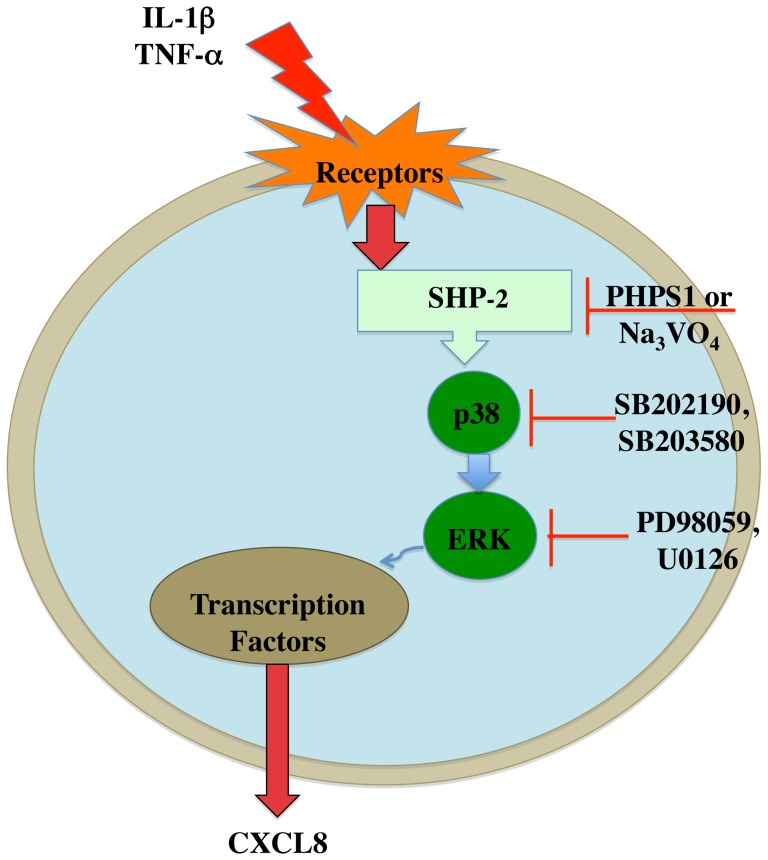
Signaling pathways involved in increased CXCL8 production in activated human astrocytes. Proinflammatory cytokines, such as interleukin (IL)-1β and tumor necrosis factor (TNF)-α, stimulate astrocytes in turn activating protein tyrosine phosphatase SHP2. Specific inhibitors are indicated adjacent to target. SHP2 is upstream of p38, which directly or indirectly modulates extracellular signal regulated kinase (ERK) mitogen-activated protein kinases activity, leading to increased expression of CXCL8 protein.

Our data indicate that IL-1β-activated astrocytes exhibit increased SHP2 phosphorylation, which correlates with the phosphorylation of p38. Furthermore, the loss of p38 and ERK activation in SHP2-deficient astrocytes implicates SHP2 as an important mediator in the p38/ERK MAPK activation. It suggests that SHP2-mediated p38 and ERK MAPK signaling regulate expression of chemokine CXCL8 in human astrocytes **(** **Figure 8** **)**. The data presented in this study provide insights into the regulation of CXCL8 in astrocytes during inflammation.

### Conclusions

Astrocytes produce CXCL8 in response to inflammatory stimuli, which is regulated by activation of SHP2 upstream to activation of p38 and ERK MAPK. The study provides insights into the signaling mechanisms of CXCL8 in human astrocytes that may be employed, in future, to therapeutically modulate CXCL8 levels *in vivo* in the context of neuroinflammation. Also, we have delineated a novel-signaling pathway in astrocytes, where phosphorylation of SHP2 is an important event in downstream signaling for production of CXCL8. Clearly, phosphorylation of SHP2 can be targeted for preventing downstream events. Future studies may be directed towards understanding SHP2 substrates in astrocytes. Also, the exact mechanisms of direct/indirect interaction of SHP2 with various docking proteins like Gab1 and Grb2 require further investigation. It will help understand how signals are transmitted downstream of IL-1β receptors, via various docking proteins to SHP2 and further down to MAPKs. Detailed understanding of astrocyte signaling will be relevant to glial-neuronal interactions, which are central to neuroinflammation in HIV-1 and many other neurodegenerative conditions.
